# Glycyrrhizin protects against porcine endotoxemia through modulation of systemic inflammatory response

**DOI:** 10.1186/cc12558

**Published:** 2013-03-11

**Authors:** Wei Wang, Feng Zhao, Yong Fang, Xiantao Li, Lei Shen, Tongwa Cao, Hechen Zhu

**Affiliations:** 1 Department of Critical Care Medicine, Huashan Hospital, Fudan University, 12 Wulumuqi Road, Shanghai, China 200040

## Abstract

**Introduction:**

Glycyrrhizin (GL) was recently found to suppress high-mobility group box 1 (HMGB1)-induced injury by binding directly to it. However, the effect of GL on HMGB1 expression in endotoxemia as well as its underlying molecular mechanism remained unclear.

**Methods:**

Twenty-one pigs were divided into four groups: sham group (n = 3), control group (n = 6), ethyl pyruvate group (n = 6) and glycyrrhizin group (n = 6). Pigs were anesthetized, mechanically ventilated, monitored and given a continuous intravenous infusion of lipopolysaccharide (LPS). Twelve hours after the start of the LPS infusion, ethyl pyruvate (30 mg/kg/hr) or glycyrrhizin (1 mg/kg/hr) was administered for 12 hours. Systemic and pulmonary hemodynamics, oxygen exchange, and metabolic status were measured. The concentrations of cytokines in serum and the corresponding gene and protein expressions in tissue samples from liver, lungs, kidneys, small intestine and lymph nodes were measured.

**Results:**

GL maintained the stability of systemic hemodynamics and improved pulmonary oxygen exchange and metabolic status. GL also attenuated organ injury and decreased the serum levels of HMGB1 and other pro-inflammatory cytokines by inhibiting their gene and protein expression.

**Conclusions:**

GL improved systemic hemodynamics and protected vital organs against porcine endotoxemia through modulation of the systemic inflammatory response. By reducing the serum level and gene expression of HMGB1 and other pro-inflammatory cytokines, GL may become a potential agent for the treatment of sepsis.

## Introduction

Sepsis is a systemic host response to infection with a clinical spectrum ranging from hemodynamic changes to multiple organ dysfunction syndrome and even death, and it is the leading cause of death in surgical intensive care unit patients [1,2]. A harmful host response to bacterial infection is believed to be the key origin of sepsis, in which invading bacteria and their products such as lipopolysaccharide (LPS) are potent activators of the inflammatory reaction [3]. The pathogenesis of sepsis involves a complex process of cellular activation at multiple levels resulting in release of pro-inflammatory cytokines such as TNF-α, IL-1β, high-mobility group box 1(HMGB1) and anti-inflammatory cytokines, such as IL-10. Other mechanisms include activation of neutrophils, monocytes and microvascular endothelial cells as well as activation of the complement, coagulation and fibrinolytic systems [4,5]. Sepsis is characterized by impaired regulation of these inflammatory responses but the exact molecular mechanism is still poorly understood.

In early studies, the efficacy of anti-TNF-α and IL-1 receptor-antagonist therapies in human sepsis were disappointing, partly because TNF-α and IL-1β were released early in the development of sepsis, thereby limiting the effect of such therapies. Unlike other pro-inflammatory cytokines, HMGB1 is a late appearing inflammatory cytokine and might become a unique target for treatment in sepsis, since it provides a sufficient time frame for clinical intervention against progressive inflammatory disorders [6].

HMGB1, originally described as a ubiquitous nuclear protein, has also been shown to be a late inflammatory cytokine in sepsis. HMGB1 may be 'passively released' from damaged or necrotic cells in injured tissues or 'actively secreted' by immune cells, including macrophages and monocytes in response to infection, peaking at approximately 20 hours after stimulation [7,8]. Moreover, HMGB1 may trigger an inflammatory response and contribute to the pathological progression of infectious and inflammatory disorders, such as enhancing the production of TNF-α, IL-1β and nitric oxide [9,10].

Several strategies of HMGB1-based therapy have been studied. Neutralizing strategies for HMGB1 inhibition including 'A-box' or anti-HMGB1 antibody treatment, have successfully reduced mortality in mice with polymicrobial sepsis. This effect was presumably due to blockade of HMGB1 binding to cell surface receptors, thereby attenuating its pro-inflammatory capability [11]. In a different strategy, pharmacological agents were studied for HMGB1-inhibiting effects. Ethyl pyruvate (EP), a stable aliphatic ester used as a nontoxic food additive as well as an experimental anti-inflammatory agent, was found to specifically inhibit LPS-induced HMGB1 secretion, to decrease circulating HMGB1 levels and to inhibit p38 mitogen-activated protein kinase (MAPK) and nuclear factor-kappa B (NF-κB) activation, ultimately resulting in improved survival in a murine sepsis model [12,13]. In a phase II multicenter double-blind placebo-controlled study of EP in high-risk patients undergoing cardiac surgery with cardiopulmonary bypass (CPB), neither benefit nor side-effect was found with EP administration [14]. No clinical study of EP in sepsis patients was found and the safety and efficacy of EP need more investigation.

Herbal remedies, such as Danggui (*Angelica sinensis*) [15], Green tea (*Camellia sinensis*) [16,17], and Danshen (*Saliva miltorrhiza*) [18] have also been found to reduce mortality in septic animals by inhibiting HMGB1 secretion. Glycyrrhizinic acid or glycyrrhizin (GL) [19], a glycoconjugated triterpene extracted from the root of Leguminosae *Glycyrrhiza glabra L*. was recently found to decrease the concentration of HMGB1 by binding directly to it and, therefore, to suppress HMGB1-induced injury [20,21]. This could be partly explained by the anti-inflammatory properties of GL and might direct the future design of new derivatives with improved HMGB1-binding properties [22]. GL has many pharmacologic actions, including anti-inflammatory, anti-viral, anti-tumor and hepatoprotective activities [23-25] and has been used clinically for more than 20 years for allergic diseases and chronic hepatitis in China and Japan. Although its safety and efficacy are widely accepted, the effect of GL on HMGB1 expression in sepsis or endotoxemia as well as the underlying molecular mechanism has not been reported up to now.

In the present study, GL was administered in a porcine endotoxemic model to examine its effects on systemic hemodynamics and organ functions as well as systemic and local inflammatory reactions while EP was used as a positive control. We further hypothesized that GL could protect against porcine endotoxemia through modulation of the systemic inflammatory response by reducing the serum level and gene expression of HMGB1 and other pro-inflammatory cytokines.

## Materials and methods

### Animal preparation

The study protocol was approved by the Animal Care and Use Committee of Fudan University (Shanghai, China). Twenty-one minipigs with a median body weight of 20 kg (range, 18 to 25 kg) were fasted for 24 hours with free access to water. All experiments were performed in the Covidien Clinical Institute, Shanghai, China.

### Anesthesia

After premedication with ketamine 15 mg/kg and midazolam 0.5 mg/kg intramuscularly, an ear vein was cannulated and midazolam 0.5 mg/kg and atropine 0.02 mg/kg were given intravenously before endotracheal intubation. Anesthesia was maintained with a continuous infusion of propofol 5 to 8 mg/kg/hr and fentanyl (30 μg/kg/hr during the surgical preparation and reduced to 5 μg/kg/hr for the rest of the experiment) and midazolam 0.5 mg/kg/hr. No muscle relaxants were used. Mechanical ventilation was initiated in constant-flow, volume-cycled mode (840 ventilator, Puritan Bennett, Tyco Healthcare, Carlsbad, CA, USA), with a tidal volume (VT) of 10 to 12 ml/kg, a frequency of 15 breaths/minute, a positive end expiratory pressure (PEEP) of 5 cmH_2_O, an inspiratory to expiratory time ratio of 1:2, and an inspired oxygen fraction of 0.50. Ringer's solution was infused at 7 to 20 mL/kg/hr during surgery to maintain normovolemia.

### Catheterization and measurement

A thermodilution Swan-Ganz catheter(5F, Arrow International Inc., Reading, PA, USA) was advanced into the pulmonary artery to be used for measuring mean pulmonary artery pressure (MPAP), pulmonary artery wedge pressure (PAWP) and central venous pressure (CVP). A femoral artery catheter (18G, Braun, Melsungen, Germany) was inserted to monitor heart rate (HR) and mean arterial pressure (MAP) and to sample blood. A central venous catheter (14F, Arrow International) was inserted via the left femoral vein to infuse fluid and drugs. Cystostomy was performed and a catheter was placed to measure urine output.

Arterial blood gas analyses (i-STAT, Abbott Point of Care Inc., Princeton, NJ, USA), including pH, PaCO_2_, PaO_2_, HCO_3_, lactate, base excess (BE), and SaO_2_, were performed at data collection points. A portion of the blood sample was examined for the following hematological parameters: white blood cell (WBC), red blood cell (RBC), hemoglobin (Hb) and platelet (Plt) counts, prothrombin time (PT), alanine aminotransferase (Alt), creatinine (Cr) andserum sodium.

### Experimental protocol

The animals were randomly assigned to four groups: sham group (n = 3, 3 males, body weight 21.67 ± 2.08 kg), control (con) group (n = 6, 3 males/3 females, body weight 18.67 ± 1.15 kg), EP treatment (EP) group (n = 6, 4 males/2 females, body weight 20.00 ± 1.73 kg), GL treatment (GL) group (n = 6, 3 males/3 females, body weight 21.67 ± 2.89 kg).

**Figure 1:**
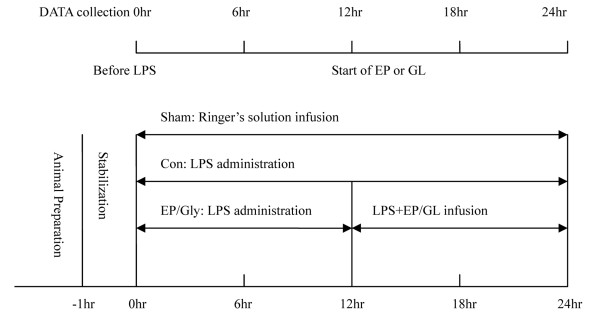
**Experimental design**. After stabilization, LPS was infused to pigs for 24 hours and after 12 hours, the EP and GL groups were treated with the corresponding drug. Systemic and pulmonary hemodynamics, gas exchange and metabolic status were measured and blood samples collected at 6, 12, 18 and 24 hours. EP, ethyl pyruvate; GL, glycyrrhizin; LPS, lipopolysaccharide.

All animals were allowed to stabilize for at least 60 minutes after surgery. After baseline data collection, the animals in the sham group were only anesthetized and infused with Ringer's solution, and the animals in the other three groups were given intravenous LPS (*Escherichia coli *0111:B4, Sigma Chemical, St. Louis, Mo, USA, 20 mg/L in 5% dextrose) infusion and Ringer's solution for 24 hours. LPS was started at 1.7 μg/kg/hr until MPAP reached 35 mmHg in 2 hours; then the infusion rate was adjusted to maintain MPAP at 35 to 45 mmHg for the remaining 22 hours. If MAP decreased below 50 mmHg despite additional intravenous fluids, the LPS infusion was temporarily suspended. After 12 hours of continuous intravenous LPS infusion, the EP group received EP (Sigma Chemical) 30 mg/kg as loading dose over 10 minutes followed by 30 mg/kg/hr for 12 hours; the GL group received GL (Minophagen Pharmaceutical Co., Tokyo, Japan) 1 mg/kg as loading dose over 10 minutes followed by 1 mg/kg/hr for 12 hours (Figure 1). The additional intravenous fluid for the four groups was as follows: sham 6.95 ± 0.79, con 9.86 ± 1.28, EP 9.62 ± 0.55, and GL 9.07 ± 0.71 ml/kg/hr. Hydroxyethylstarch (HAES-Steril^® ^6% 200/0.5, Fresenius Kabi, Erlangen, Germany) was administered as required to maintain MAP > 60 mmHg, and 5% glucose solution was infused to keep arterial blood glucose levels between 5 to 7 mmol/L [1,26]. Con, EP and GL groups received similar amount of HAES (con, 64.0 ± 8.19, EP, 67.33 ± 6.66, and GL, 63.33 ± 4.93 ml).

Systemic and pulmonary hemodynamics, oxygen exchange, and metabolic parameters were measured 6 hours, 12 hours (that is, immediately before EP or GL), 18 hours and 24 hours after the start of LPS infusion. Blood samples were collected at 6, 12, 18 and 24 hours after the start of LPS infusion. During the experiment, body temperature was maintained between 37.5 and 38.5°C using external cooling and heating pads. After completion of the experiment, the animals were sacrificed by potassium chloride injection under deep anesthesia.

Tissue specimens from lung, liver, kidney, small intestine and lymph node were aseptically harvested and put into 10% formalin for pathological examination (H & E stain) or snap frozen in liquid nitrogen (-180°C) and then stored at -80°C for western-blot and real-time PCR.

### Histopathology

After H & E stain, at least six random areas (× 200) from each section were analyzed using a scoring system [see Additional file 1] [27]. The lung was assessed for leukocyte infiltration, hemorrhage and alveolar wall thickness. Liver samples were assessed for interstitial edema and leukocyte infiltration. The kidney was assessed for interstitial edema, leukocyte infiltration and capillary congestion. The small intestine was assessed for Gruenhagen's spaces, denuded surface and interstitial edema.

### Cytokine measurements

Concentrations of HMGB1, IL-1β, TNF-α, IL-6 and IL-10 in serum samples were determined simultaneously by using a commercial ELISA kit (HMGB1, IBL International GmbH, Hamburg, Germany; TNF-α, R&D Systems, Minneapolis, MN, USA; IL-6, antibodies-online Inc., Atlanta, GA, USA; and IL-1β, IL-10, ALPCO Diagnostics, Salem, NH, USA). Samples and standards were prepared following the manufacturer's instructions and analyzed on a microplate reader (Model 680, Bio-Rad Laboratories, Inc., Hercules, CA, USA). The detection limits were 0.5 ng/ml for HMGB1, 10 pg/ml for IL-1β, 10 pg/ml for TNF-α, 50 pg/ml for IL-6 and 10 pg/ml for IL-10. The intra-assay variations were 2.8% to 3.4% for HMGB1, 3.4% to 6.8% for IL-1β, 4.3% to 6.9% for TNF-α, 3.5% to 5.9% for IL-6 and 3.2% to 6.0% for IL-10. The inter-assay variations were 3.8% to 6.4%, 8.4% to 9.8%, 7.3% to 9.9%, 6.5% to 11.9% and 5.3% to 8.0% for the same cytokines, respectively.

### Diamine oxidase and D-lactate Assay

Concentrations of diamine oxidase (DAO) and D-lactate in serum samples were determined by using a DAO ELISA kit (E0656p, EIAab Science Co., Wuhan, China) or D-lactate Colorimetric Assay Kit (K667-100, BioVision, Inc., Milpitas, CA, USA). Samples and standards were prepared following the manufacturer's instructions and analyzed on a microplate reader (Model 680, Bio-Rad Laboratories, Inc.). The detection limits were 0.5 U/ml for DAO and 0.01 nmol/μl for D-lactate. The intra-assay variations were 3.0% to 6.2% for DAO and 3.2% to 6.0% for D-lactate. The inter-assay variations were 8.8% to 11.9% and 6.6% to 10.0% for DAO and D-lactate, respectively.

### Western-blot

Frozen tissue samples were pulverized and homogenized in a homogenization buffer (10 mmol/L phosphate buffer, 250 mmol/L sucrose, 1 mmol/L ethylenediaminetetraacetic acid (EDTA), 0.1 mmol/L phenylmethanesulfonyl fluoride (PMSF) and 0.1% tergitol, pH 7.5). Homogenates were centrifuged at 27 000*g *for 10 minutes at 4°C and supernatant as total protein extracts were isolated. Nuclear protein was extracted following the manufacturer's instructions using a nuclear extract kit (40010 & 40410, Active Motif, Carlsbad, CA, USA) to determine NF-κB p65. Protein extracts were resolved on 12% SDS polyacrylamide gel and transferred to nitrocellulose membranes. The membranes were probed overnight at 4°C with the following primary antibodies: antibody against HMGB1 (LS-B2841) (LifeSpan Biosciences, Inc., Seattle, WA, USA), IL-10 (sc-74141), VCAM-1(vascular cell adhesion molecule 1, sc-73252) (Santa Cruz Biotechnology, Inc., Santa Cruz, CA., USA), antibody against IL-6 (ab11746), antibody against β-actin (ab3280) (Abcam plc., Cambridge, MA, USA), antibody against NF-κB p65 (ABIN121274) (antibodies-online Inc.), antibody against ICAM-1(intercellular adhesion molecule-1, Boster Biological Technology, Ltd., Wuhan, China) and the secondary antibody (sc-358914, sc-2004, sc-2006)(Santa Cruz Biotechnology, Inc.), respectively.

Antibodies were diluted to a final concentration of 0.5 to 2 μg/ml. The protein bands were visualized by a chemiluminescence imaging system (ChemiDoc™ XRS, Bio-Rad Laboratories, Inc.). The signal intensity was quantified by ImageJ 1.44 software. Densitometric analysis of the expression levels of specific proteins on the immunoblots was normalized to that of β-actin. Representative immunoblots from five independent experiments are shown.

### Real-time PCR

Total RNA was extracted by using the RNeasy Mini Kit (Qiagen, Hilden, Germany). Contaminating DNA was removed using RNase-Free DNase Set (Qiagen). cDNA was generated from 1 μg total RNA, and 1/20th of the cDNA mixture was used for quantitative reverse transcription in the 7300 Real-time PCR System (ABI, Carlsbad, CA, USA). Approximately 25 μl of reaction mixture was used for the real-time PCR assay that consisted of 2 × (12.5 μl) SYBR green supermix (TIANGEN BIOTECH CO., Beijing, China), 1 μl primers (0.5 μl each from the stock), 11 μl of water, and 0.5 μl of template. The thermal conditions consisted of an initial denaturation at 95°C for 10 minutes followed by 40 cycles (30 seconds at 95°C, 30 seconds at 58°C, 30 seconds at 72°C). The sequences of primers used are listed in Additional file 2, Table S1. All reactions were carried out in triplicate to reduce variation.

The data were analyzed using ABI Prism 7300 SDS software. Data normalization was accomplished using the endogenous control (β-actin) and the normalized values were subjected to a 2^-ΔΔ*Ct *^formula to calculate the fold change between the control and experiment groups.

### Statistical analysis

All data are expressed as mean ± SD. Comparison between groups was performed by repeated measurements analysis of variance (ANOVA) for the data as continuous variables (that is, systemic and pulmonary hemodynamics, oxygen exchange and metabolic data), followed by post hoc testing with Fisher's protected least significant difference test. For other data, differences within each group were tested using a two way ANOVA and a subsequent Student-Newman-Keuls for multiple comparisons. In all cases, *P *< .05 was regarded as statistically significant.

## Results

### Systemic and pulmonary hemodynamics, oxygen exchange and metabolic data

Con, EP and GL groups received similar LPS infusions (con, 1.23 ± 0.27; EP, 1.37 ± 0.21; GL, 1.30 ± 0.24 μg/kg/hr). Systemic and pulmonary hemodynamics, oxygen exchange and metabolic data are summarized in Table 1.

**Table 1:** Systemic and pulmonary hemodynamics, oxygen exchange and metabolic data.

	Baseline	6 hours	12 hours	18 hours	24 hours
pH					
Sham	7.47 ± 0.01	7.47 ± 0.01	7.47 ± 0.01	7.46 ± 0.04	7.46 ± 0.02
Con	7.47 ± 0.01	7.42 ± 0.02	7.41 ± 0.03	7.41 ± 0.03	7.44 ± 0.03
EP	7.48 ± 0.03	7.46 ± 0.03	7.39 ± 0.03	7.38 ± 0.02	7.40 ± 0.04
GL^ab^	7.57 ± 0.02	7.41 ± 0.09	7.51 ± 0.14	7.40 ± 0.05	7.38 ± 0.07
PaCO_2 _(mmHg)					
Sham	38.8 ± 1.9	38.3 ± 3.2	39.3 ± 2.8	40.2 ± 2.8	39.0 ± 3.2
Con	40.9 ± 1.8	43.9 ± 2.7	42.6 ± 2.3	42.9 ± 2.6	41.9 ± 2.6
EP	37.2 ± 3.0	37.9 ± 4.8	36.5 ± 1.8	36.4 ± 2.5	36.3 ± 2.0
GL	36.1 ± 2.0	36.5 ± 11.9	35.6 ± 10.7	43.2 ± 5.4	40.8 ± 5.9
PaO_2 _(mmHg)					
Sham	251.7 ± 16.3	234.7 ± 19.4	231.3 ± 32.3	240.3 ± 17.6	230.0 ± 19.5
Con^a^	255.3 ± 13.9	168.3 ± 16.9	170.0 ± 19.5	181.0 ± 14.6	189.3 ± 23.3
EP^ab^	254.3 ± 12.2	164.3 ± 17.4	175.7 ± 14.2	223.7 ± 20.0	221.0 ± 34.1
GL^ab^	257.7 ± 13.4	161.3 ± 22.3	172.7 ± 13.9	231.3 ± 22.2	229.0 ± 24.8
HCO_3 _(mmHg)					
Sham	29.5 ± 2.7	28.3 ± 1.8	29.1 ± 2.2	30.2 ± 3.0	29.0 ± 2.5
Con	32.1 ± 3.1	30.6 ± 1.7	30.1 ± 1.3	29.0 ± 1.5	30.2 ± 1.8
EP^ab^	29.8 ± 3.4	27.0 ± 1.4	23.8 ± 1.1	23.4 ± 1.4	23.9 ± 1.6
GL	33.3 ± 2.1	33.0 ± 6.7	29.3 ± 5.6	30.3 ± 5.1	33.5 ± 4.5
BE (mmol/L)					
Sham	3.7 ± 1.5	2.7 ± 1.2	3.3 ± 2.1	1.7 ± 0.6	1.7 ± 1.5
Con^a^	4.0 ± 3.1	0.3 ± 2.7	-2.7 ± 2.1	-2.0 ± 1.5	-3.3 ± 2.3
EP^ab^	4.3 ± 1	1.0 ± 0.9	-2.7 ± 1.0	-1.0 ± 0.9	1.0 ± 0.6
GL^ab^	4.0 ± 1.3	0.3 ± 0.8	-1.7 ± 1.4	-0.3 ± 1.4	1.5 ± 0.5
Lactate (mmol/L)					
Sham	0.8 ± 0.4	0.9 ± 0.4	1.0 ± 0.5	0.9 ± 0.4	1.0 ± 0.5
Con^a^	0.9 ± 0.4	1.9 ± 0.6	2.6 ± 0.8	2.4 ± 0.1	2.3 ± 0.2
EP^ab^	1.3 ± 0.4	2.1 ± 0.6	2.7 ± 0.8	1.3 ± 0.4	1.1 ± 0.3
GL^ab^	1.2 ± 0.6	2.5 ± 1.0	3.3 ± 1.4	1.4 ± 0.9	1.0 ± 0.5
HR (beats/min)					
Sham	126.7 ± 7.0	123.0 ± 16.6	121.0 ± 11.5	123.0 ± 7.9	129.3 ± 4.0
Con^a^	135.0 ± 13.4	89.3 ± 8.5	90.0 ± 7.2	97.7 ± 7.2	102.3 ± 8.7
EP^a^	125.0 ± 11.8	88.3 ± 14.9	88.0 ± 6.3	100.3 ± 4.9	109.0 ± 4.5
GL^a^	123.0 ± 10.1	87.7 ± 8.3	92.7 ± 9.6	102.3 ± 7.2	114.7 ± 9.0
MAP (mmHg)					
Sham	94.3 ± 8.02	95.3 ± 4.7	87.7 ± 8.5	85.7 ± 6.0	82.7 ± 6.8
Con^a^	89.3 ± 7.2	67.7 ± 12	64.3 ± 7.6	65.7 ± 9.6	67.0 ± 12.6
EP^ab^	82.7 ± 8.3	65.7 ± 11.5	63.7 ± 7.4	72.7 ± 9.6	84.3 ± 9.3
GL^ab^	84.3 ± 11.5	63.7 ± 5.1	60.0 ± 5.6	76.3 ± 5.8	85.7 ± 4.6
MPAP (mmHg)					
Sham	20.7 ± 4.7	22.7 ± 7.2	26.3 ± 6.8	25.3 ± 5.0	26.3 ± 6.4
Con	19.3 ± 1.4	39.3 ± 7.6	37.3 ± 4.4	38.7 ± 2.7	38.0 ± 2.4
EP	19.7 ± 4.6	37.7 ± 3.6	37.7 ± 2.7	33.0 ± 2.4	30.7 ± 2.1
GL	20.3 ± 4.6	37.0 ± 3.2	38.7 ± 2.6	36.7 ± 1.0	31.3 ± 4.4
CVP (mmHg)					
Sham	11.0 ± 2.0	10.3 ± 0.6	12.3 ± 3.8	15.3 ± 2.5	12.3 ± 2.3
Con	11.7 ± 2.1	11.3 ± 0.5	13.3 ± 2.6	11.7 ± 3.7	10.3 ± 1.9
EP	9.0 ± 2.4	9.3 ± 4.2	11.3 ± 4.5	11.0 ± 5.0	12.3 ± 3.4
GL	13.3 ± 2.3	14.0 ± 4.7	12.7 ± 4.6	13.0 ± 3.9	13.7 ± 2.9
PAWP (mmHg)					
Sham	8.0 ± 1.7	7.3 ± 1.5	6.7 ± 1.2	7.3 ± 0.6	7.3 ± 1.5
Con	7.0 ± 1.8	7.3 ± 2.6	8.3 ± 3.4	8.0 ± 3.2	7.0 ± 0.9
EP	8.7 ± 3.1	8.0 ± 2.4	7.0 ± 0.9	9.3 ± 1.4	7.3 ± 1.4
GL	7.7 ± 1.4	6.7 ± 1.9	6.0 ± 0.9	6.3 ± 1.9	7.7 ± 1.4
Urine (ml/kg/h)					
Sham	0	5.0 ± 0.5	6.4 ± 1.1	6.1 ± 1.0	5.1 ± 1.0
Con	0	6.4 ± 1	6.0 ± 1.8	6.0 ± 1.5	5.5 ± 0.8
EP^ab^	0	6.1 ± 0.9	7.5 ± 1.1	7.7 ± 1.0	8.5 ± 1.1
GL^ab^	0	5.1 ± 0.8	6.7 ± 0.8	7.4 ± 1.0	8.0 ± 0.6

^a^*P *< 0.05 versus sham group by repeated measures ANOVA; ^b^*P *< 0.05 versus Con group by repeated measures ANOVA. Data are mean ± SD. ANOVA, analysis of variance; BE, base excess; Con, control; CVP, central venous pressure; EP, ethyl pyruvate; GL, glycyrrhizin; HR, heart rate; MAP, mean arterial pressure; MPAP, mean pulmonary artery pressure; PAWP, pulmonary artery wedge pressure.

There were no differences between groups in baseline hemodynamics (HR, MAP, MPAP, CVP and PAWP), which remained unaltered throughout the experiment in the sham group (*P *> .05). LPS infusion increased MPAP (*P *< .05), whereas MAP decreased compared to the sham group (*P *< .05). Both CVP and PAWP remained stable throughout the experiment without intergroup difference.

There were no differences between groups in arterial pH, PaCO_2 _and HCO_3_, whereas PaO_2 _significantly decreased as a result of LPS infusion. EP and GL increased PaO_2_, which meant the oxygen exchange was improved in the EP and GL groups.

### Effect of GL on organ injury in endotoxemic pigs

As shown in Table 2 GL and EP markedly reversed the increase of WBC and decrease of Plt induced by LPS. Cr in the GL or EP group was significantly lower than in the con group, although Cr in all groups increased compared with baseline.

**Table 2:** Blood cell count, chemistry and organ functions.

	Baseline	6 hours	12 hours	18 hours	24 hours
WBC (×10^9^/L)					
Sham	11.8 ± 2.1	11.6 ± 1.4	13.1 ± 1.4	15.0 ± 2.4	15.1 ± 1.5
Con^a^	9.3 ± 0.4	3.2 ± 0.5	3.8 ± 0.5	17.2 ± 0.3	22.0 ± 3.9
EP^ab^	9.7 ± 1.9	3.3 ± 0.9	3.2 ± 0.6	11.3 ± 1.8	13.3 ± 2.7
GL^ab^	9.5 ± 3.7	3.4 ± 1.3	4.3 ± 0.8	9.0 ± 1.8	13.5 ± 5.9
Plt (×10^12^/L)					
Sham	278.7 ± 10.4	291.7 ± 14.6	292.7 ± 11.0	276.3 ± 11.1	273.7 ± 17.6
Con^a^	274.7 ± 39.8	158.7 ± 21.3	64.0 ± 3.1	57.3 ± 9.0	53.7 ± 9.1
EP^ab^	316.7 ± 48.8	139.0 ± 14.2	68.0 ± 9.1	169.3 ± 40.4	185.0 ± 7.6
GL^ab^	343.3 ± 45.2	165.0 ± 20.1	85.0 ± 18.5	184.3 ± 15.8	172.0 ± 16.3
Alt (mmol/L)					
Sham	52.7 ± 6.4	55.3 ± 4.5	55.3 ± 8.4	55.7 ± 3.2	57.7 ± 5.0
Con^a^	59.3 ± 4.8	71.0 ± 8.3	69.0 ± 7.0	75.7 ± 12.5	75.0 ± 8.9
EP^a^	56.3 ± 5.9	68.0 ± 5.7	72.0 ± 8.0	73.3 ± 8.1	74.3 ± 7.6
GL^ab^	59.0 ± 5.5	71.3 ± 5.4	72.8 ± 5.6	57.7 ± 4.1	56.0 ± 6.4
Cr (mmol/L)					
Sham	71.3 ± 8.4	77.0 ± 9.6	79.7 ± 9.0	83.0 ± 6.6	88.3 ± 5.8
Con^a^	70.0 ± 3.9	83.7 ± 6.9	119.7 ± 6.7	181.3 ± 14.5	211.0 ± 21.7
EP^ab^	77.7 ± 6.6	81.0 ± 4.6	109.3 ± 5.1	146.0 ± 33.1	180.7 ± 11.9
GL^ab^	74.7 ± 12.1	82.0 ± 23.3	111.7 ± 27.9	116.7 ± 21.2	143.7 ± 15.9
PT (seconds)					
Sham	11.2 ± 1.2	12.0 ± 1.9	12.0 ± 1.6	11.9 ± 1.9	12.4 ± 2.4
Con^a^	11.2 ± 1.6	12.1 ± 1.6	14.8 ± 2.3	14.9 ± 1.7	14.4 ± 2.2
EP^a^	10.4 ± 0.5	12.1 ± 1.3	14.3 ± 1.9	15.4 ± 1.3	15.1 ± 1.5
GL^a^	10.9 ± 0.8	12.6 ± 1.3	14.4 ± 0.7	14.8 ± 1.2	14.9 ± 1.3
Na (mmol/L)					
Sham	140.7 ± 1.2	141.3 ± 1.5	143.7 ± 2.3	144.0 ± 1.0	144.7 ± 1.2
Con^a^	140.7 ± 3.6	141.7 ± 1.4	140.7 ± 2.3	139.3 ± 2.9	140.7 ± 2.3
EP	144.0 ± 0.9	143.0 ± 0.9	144.0 ± 0.9	139.3 ± 5.7	143.0 ± 2.7
GL^b^	145.3 ± 2.3	145.7 ± 2.9	143.0 ± 0.9	143.3 ± 3.6	143.7 ± 3.6

^a^*P *< 0.05 versus sham group by repeated measures ANOVA; ^b^*P *< 0.05 versus Con group by repeated measures ANOVA; Data are mean ± SD. Alt, alanine aminotransferase; ANOVA, analysis of variance; Con, control; Cr, creatinine; EP, ethyl pyruvate; GL, glycyrrhizin; Plt, platelet; PT, prothrombin time; WBC, white blood cell.

**Figure 2:**
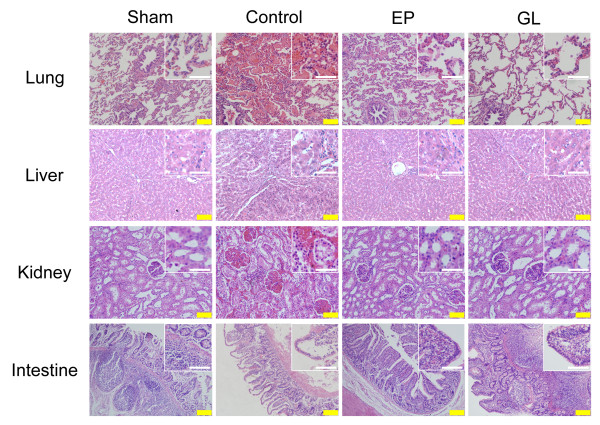
**Organ injury in each group [hematoxylin and eosin (H&E), yellow bar = 100 μm, white bar = 50 μm]**. Lung: A marked infiltration of inflammatory cells and hemorrhage were seen in the control group. The EP and GL groups had slighter leukocyte infiltration and almost no hemorrhage. Liver: The control group was marked by interstitial edema. The EP group had a similar change but to a lesser extent. Kidney: The control group had significant interstitial edema and capillary congestion. The EP and GL groups had slighter edema and almost no capillary congestion. Intestine: Severe edema was observed in the small intestine of the control group. The EP and GL groups had similar change but to a lesser extent. EP, ethyl pyruvate; GL, glycyrrhizin.

Microscopy of lung tissue showed that LPS caused a marked infiltration of inflammatory cells and hemorrhage in the con group compared to the sham group, whereas such changes were obviously attenuated in the GL group (Figure 2, Table 3). In the liver, EP and GL led to significant decreases in interstitial WBCs. The sections of the kidney showed a significant decrease in interstitial edema, leukocyte infiltration and capillary congestion when EP and GL were used. EP and GL also caused a significant decrease of Gruenhagen's spaces, denuded surface and interstitial edema in the small intestine (Figure 2, Table 3). There was no obvious injury in the lymph nodes in each group.

**Table 3:** Histological score.

Histological change	Sham	Con	EP	GL
Lung				
Leukocyte infiltration	0.92 ± 0.32	2.85 ± 0.61^a^	1.92 ± 0.36^a^	1.7 ± 0.36^ab^
Hemorrhage	0.7 ± 0.13	3.14 ± 0.57^a^	1.52 ± 0.12^ab^	1.65 ± 0.56^ab^
Alveolar wall thickness	0.92 ± 0.32	1.78 ± 0.26^a^	1.74 ± 0.68	1.36 ± 0.47
Liver				
Interstitial edema	0.77 ± 0.33	1.86 ± 0.32^a^	1.37 ± 0.16^ab^	1.65 ± 0.3^a^
Leukocyte infiltration	0.33 ± 0.27	2.64 ± 0.48^a^	1.67 ± 0.46^ab^	1.36 ± 0.32^ab^
Kidney				
Interstitial edema	1.3 ± 0.19	2.32 ± 0.35^a^	1.65 ± 0.14^ab^	1.64 ± 0.19^ab^
Leukocyte infiltration	0.64 ± 0.15	3.01 ± 0.34^a^	2.67 ± 0.32^ab^	2.11 ± 0.19^ab^
Capillary congestion	0.24 ± 0.17	3.12 ± 0.13^a^	1.96 ± 0.22^ab^	1.69 ± 0.18^ab^
Small intestine				
Gruenhagen's spaces	0.67 ± 0.13	2.36 ± 0.34^a^	1.65 ± 0.35^ab^	1.32 ± 0.28^ab^
Denuded Surface	0.89 ± 0.32	2.17 ± 0.12^a^	1.35 ± 0.32^b^	1.41 ± 0.16^ab^
Interstitial edema	0.4 ± 0.24	3.15 ± 0.37^a^	1.98 ± 0.27^ab^	1.31 ± 0.17^ab^

^a^*P *< 0.05 versus sham group, ^b^*P *< 0.05 versus Con group. Con, control; EP, ethyl pyruvate; GL, glycyrrhizin.

**Figure 3:**
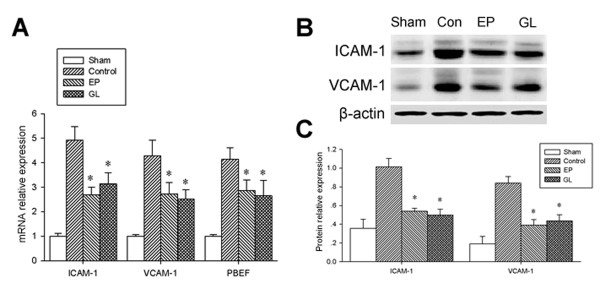
**mRNA expression of ICAM-1, VCAM-1 and PBEF and protein expression of ICAM-1 and VCAM-1 in lung**. **A**) Real-time PCR showed that the expression levels of ICAM-1, VCAM-1 and PBEF mRNA decreased significantly in the EP and GL groups compared to the control group. **B**) Western-blot showed that the expression levels of ICAM-1 and VCAM-1 protein decreased significantly in the EP and GL groups compared to the control group. **C**) Quantitative assessment of protein relative to β-actin showed that the expression levels of ICAM-1 and VCAM-1 protein decreased significantly in the EP and GL groups compared to the control group. * *P *< 0.05 versus the control group. EP, ethyl pyruvate; GL, glycyrrhizin; ICAM-1, intercellular adhesion molecule-1; PBEF, pre-B-cell colony-enhancing factor; VCAM-1, vascular cell adhesion molecule 1.

**Figure 4:**
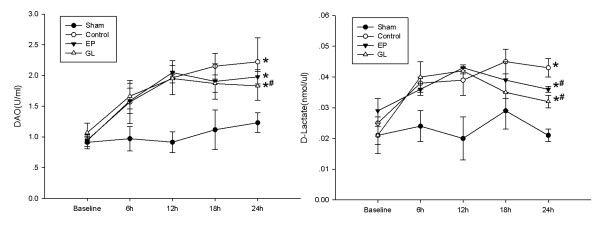
**Concentration of diamine oxidase and D-lactate in plasma**. Diamine oxidase and D-lactate in plasma increased in the control group and decreased after EP or GL was used. *P < 0.05 versus sham group by repeated measures ANOVA; # *P *< 0.05 versus con group by repeated measures ANOVA. ANOVA, analysis of variance; con, control; EP, ethyl pyruvate; GL, glycyrrhizin.

The RNA transcripts of ICAM-1, VCAM-1 and PBEF (pre-B-cell colony-enhancing factor) in the lung were significantly up-regulated by LPS compared to the sham group, whereas treatment with GL or EP reduced the extent of such up-regulation (Figure 3A). The protein of ICAM-1 and VCAM-1 in the lung showed changes similar to mRNA in the four groups (Figure 3B, C). As indicators of small intestine injury, DAO and D-lactate in serum increased due to LPS, and GL attenuated such responses with DAO and D-lactate while EP affected D-lactate only (Figure 4).

### Effect of GL on serum HMGB1, IL-1β, TNF-α and IL-10 levels

**Figure 5:**
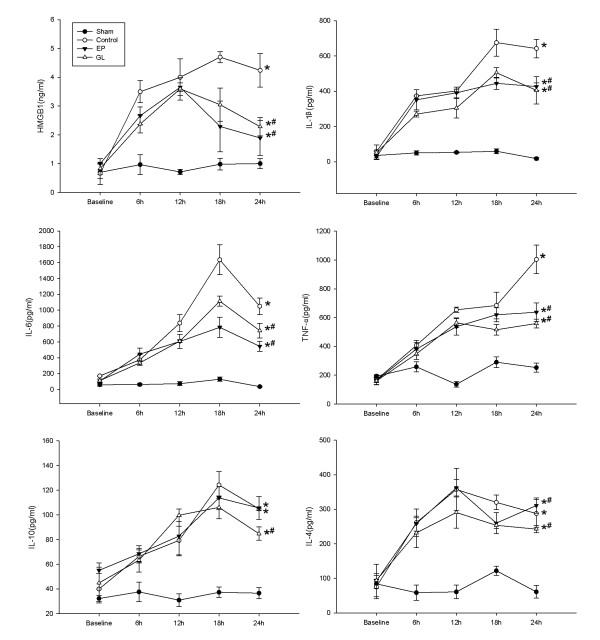
**Concentration of plasma cytokine**. *P < 0.05 versus sham group by repeated measures ANOVA; # *P *< 0.05 versus con group by repeated measures ANOVA. ANOVA, analysis of variance; con, control.

Serum cytokine concentrations in the four groups are shown in Figure 5. HMGB1 levels in serum were significantly elevated in the con, GL and EP groups compared with the sham group but were reduced after GL or EP administration.

Endotoxemia induced a characteristic cytokine response with high levels of key inflammatory cytokines, such as IL-1β, TNF-α and IL-6, but EP and GL significantly attenuated such responses compared with the con group. An increase in IL-10 induced by LPS was also detected but the peak of IL-10 appeared later than that of the other inflammatory cytokines. The IL-10 level was not affected by EP significantly. The IL-4 level began to decline after 12 hours of LPS infusion, and GL or EP further enhanced the trend.

### Effect of GL on HMGB1, IL-1β, TNF-α cytokines and NF-κB gene and protein expression

**Figure 6:**
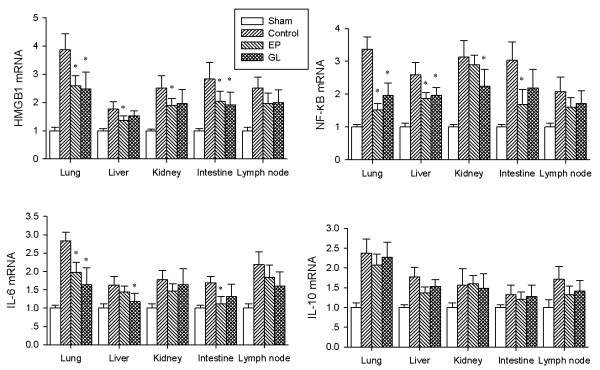
**mRNA expression of HMGB1, NF-κB p65, IL-6 and IL-10**. Realtime PCR showed the expression levels of HMGB1, NF-κB p65 (NF-κB), IL-6 and IL-10 mRNA in lung, liver, kidney, intestine and lymph node. * *P *< 0.05 versus control group. HMBG1, high-mobility group box 1; NK-κB, nuclear factor-kappaB.

**Figure 7:**
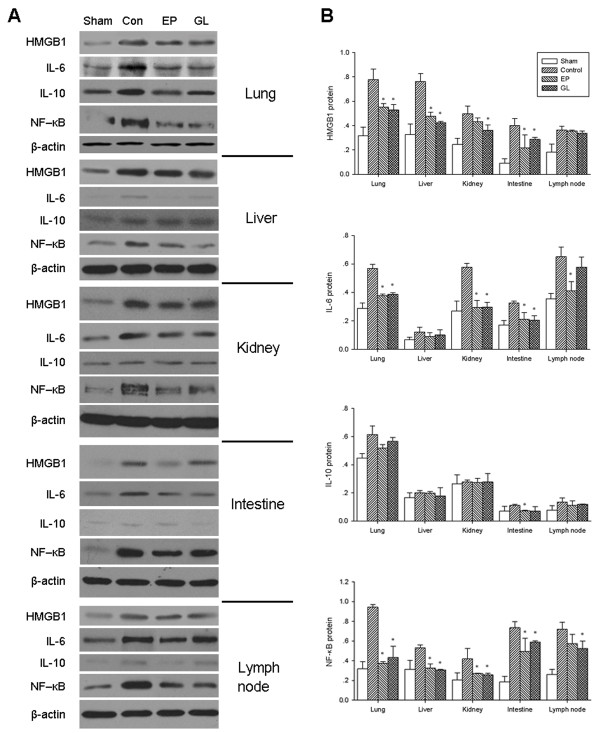
**Protein expression of HMGB1, NF-κB p65, IL-6 and IL-10**. **A**) Western-blot showed the expression levels of HMGB1, NF-κB p65 (NF-κB), IL-6 and IL-10 in lung, liver, kidney, intestine and lymph node. The expression of NF-κB p65 was detected using nuclear protein while others were detected using total protein. **B**) Quantitative assessment of protein relative to β-actin. * *P *< 0.05 versus control group. HMBG1, high-mobility group box 1; NK-κB, nuclear factor-kappaB.

As shown in Figures 6 and 7, expression levels of HMGB1 mRNA and protein in lungs, liver, kidneys, small intestine and lymph node were enhanced markedly after endotoxemia compared with the sham group. Treatment with GL or EP reduced expression levels of HMGB1 mRNA and protein in the five organs, especially in the lung and small intestine compared with the con group. Endotoxemia also up-regulated the expression of NF-κB p65 mRNA and increased activated protein of NF-κB p65 in the five organs compared with the sham group. The levels of NF-κB p65 mRNA and activated protein in most of the organs were markedly lower in the GL or EP group than the con group.

Compared with the con group, GL markedly down-regulated the expression of IL-6 mRNA in the lung and liver, but did not significantly alter the expression IL-10 mRNA (Figure 6). In the lung, kidney and intestine, EP or GL significantly reduced the level of IL-6 protein, but again, GL did not reduce the level of IL-10 protein in any of the five organs (Figure 7).

## Discussion

In the present study, a porcine model of endotoxemia was chosen because the effect of continuous low-dose LPS simulated the systemic response of endotoxemia, therefore a better observation of systemic and pulmonary hemodynamic changes and vital organ function could be made. As a positive control, EP maintained the stability of systemic hemodynamics and protected vital organ functions. More importantly, EP protected against porcine endotoxemia through modulation of systemic and local inflammatory responses.

The main findings of this study were that, following LPS treatment: 1) GL could better maintain the stability of systemic hemodynamics and improve pulmonary oxygen exchange and metabolic status. 2) GL might protect organ function by regulating excessive inflammatory reactions. 3) GL inhibited the gene and protein expression of HMGB1 and other pro-inflammatory cytokines and decreased their concentrations which might result from the inhibition of NF-κB p65.

GL stabilized the systemic hemodynamics and improved pulmonary oxygen exchange which achieved the effect similar to that of EP. We also found an obvious decrease in PaO_2 _after infusion of LPS but a distinct increase in PaO_2 _after the administration of GL, suggesting improved pulmonary oxygenation and systemic acidosis as reflected by the change of blood lactate and BE. Pathological examination revealed improvement in alveolar hemorrhage, infiltration of inflammatory cells and interstitial pulmonary edema, which was in line with other experimental findings.

Our data indicated that GL protected the lung function, not only improving the oxygenation of the lungs, but also decreasing the expression of ICAM-1, VCAM-1 and PBEF in the lungs significantly. DAO and D-lactate have been used as novel markers of intestinal ischemia and bacterial infection in the intestine [28,29]. In the present study, a significant increase in DAO and D-lactate serum levels in the con group indicated marked intestinal injury, while GL decreased the levels of DAO and D-lactate and attenuated intestinal injury. Although pseudo-aldosteronism, with sodium retention, hypokalaemia and hypertension, is a well-known side-effect of GL [30], no obvious electrolyte disturbance due to GL was found in our experiment. Overall, our study indicated that GL improved cardiovascular, respiratory and other vital organ functions during endotoxemia which resulted in protection against damage to multiple organs.

Regulation of the excessive inflammatory reactions might be a major mechanism by which GL exerted its protective effect. In the porcine model, we observed that serum and protein levels of both HMGB1 and other pro-inflammatory cytokines in the liver, lungs, kidneys, small intestine and lymph nodes increased significantly after LPS infusion, while treatment with GL reduced their serum levels in those organs. A significant reduction of HMGB1 protein in these organs except lymph nodes was observed (Figure 7), but interestingly, the mRNA level of HMGB1 was down-regulated only in the lungs and small intestine (Figure 6). It might be related to the HMGB1-binding characteristics of GL [21], which might accelerate the degradation of HMGB1 protein.

Several mechanisms have been proposed for the actions of GL: (1) it inhibited glucocorticoid metabolism and potentiated their effects [31,32]; (2) it exerted its effects through the influence of cytokines and chemokines (TNF-α, IL-1β, IFN-γ, IL-10, Eotaxin-1, etc.), ICAM-1, P-selectin and some enzymes like inducible nitric oxide synthase [33-35]; (3) it suppressed LPS-induced activation of signaling cascades and the expression of pro-inflammatory genes through the inhibition of NF-κB and PI3K activity [36,37].

IL-10 has various functions and is considered the most important anti-inflammatory cytokine in maintaining immune system balance. IL-10 could inhibit the activation of macrophages, monocytes and T cells, and limit the secretion of pro-inflammatory cytokines, such as TNF-α, IL-1, IL-6, and IL-12 [38]. Our data indicated that IL-10 serum levels and gene expression showed no significant difference between the GL group and the con group. IL-10 may inhibit the excessive inflammatory reaction more efficiently, because IL-10 levels did not decrease while TNF-α and HMGB1 levels decreased at the same time. The exact mechanism needs further investigation.

NF-κB plays a key role in the initiation and progression of inflammatory reactions. The transcription factor NF-κB has been shown to be a major regulator of many functionally diverse pro-inflammatory cytokines [39]. Active NF-κB promotes the gene expression of pro-inflammatory cytokines, such as TNF-α, IL-1β and HMGB1, adhesion molecules and chemokines [40]. Menegazzi [34] demonstrated that GL decreased the levels and gene expression of TNF-α and IL-1β in the pleural exudates and lung tissues, and the inhibition effect might be associated with the prevention of NF-κB and STAT-3 activation. We found that GL inhibited NF- κB gene expression (liver, lungs and kidneys) and NF- κB activation (liver, lungs, kidneys, small intestine and lymph nodes). At the same time, it decreased the serum levels of TNF-α, IL-1β and HMGB1, and exerted a protective effect against the damage related to pro-inflammatory cytokines. These results imply that GL inhibits the gene expression of such cytokines by inhibiting NF-κB expression and activation, but the exact molecular mechanism is unknown. Receptor for advanced glycation end products (RAGE), a major membrane receptor for HMGB1, has been shown to mediate activation of NF-κB [41] and positively regulate NF-κB p65 expression [42]. This suggests that HMGB1 and NF-κB may form a positive feedback loop and GL may modulate the excessive inflammatory and immune responses through its HMGB1-binding capability. However, the underlying molecular mechanism and interaction still awaits further exploration.

In this porcine model of endotoxemia, GL improved systemic hemodynamics, enhanced pulmonary oxygen exchange and protected against damage to multiple organs. These effects could be attributed to its regulation of excessive inflammatory and immune responses by reducing serum levels and gene expression of HMGB1 and other pro-inflammatory cytokines. Therefore, GL could protect against porcine endotoxemia through modulation of systemic inflammatory response, and GL might become a potential agent for the treatment of sepsis in the future. Further basic and clinical investigations are warranted in this area.

## Conclusions

GL could improve systemic hemodynamics and protect vital organs against porcine endotoxemia through modulation of the systemic inflammatory response, and GL might become a potential agent for the treatment of sepsis.

## Key messages

• GL could maintain the stability of systemic hemodynamics, improve pulmonary oxygen exchange and metabolic status in porcine endotoxemia.

• GL attenuated organ injury induced by LPS.

• GL decreased the serum levels of HMGB1 and other pro-inflammatory cytokines through inhibiting their gene and protein expression.

## Abbreviations

Alt, alanine aminotransferase; ANOVA, analysis of variance; BE, base excess; Con, control; CPB, cardiopulmonary bypass; Cr, creatinine; CVP, central venous pressure; DAO, diamine oxidase; EDTA, ethylenediaminetetraacetic acid; ELISA, enzyme-linked immunosorbent assay; EP, ethyl pyruvate; GL, glycyrrhizin; H & E, hematoxylin and eosin; HAES, hydroxyethyl starch; Hb, hemoglobin; HMGB1, high-mobility group box 1; HR, heart rate; ICAM-1, intercellular adhesion molecule-1; IL, interleukin; LPS, lipopolysaccharide; MAP, mean arterial pressure; MAPK, mitogen-activated protein kinase; MPAP, mean pulmonary artery pressure; NF-κB, nuclear factor-kappaB; PAWP, pulmonary artery wedge pressure; PBEF, pre-B-cell colony-enhancing factor; PCR, polymerase chain reaction; PEEP, positive end expiratory pressure; Plt, platelet; PT, prothrombin time; RBC, red blood cells; TNF-α, tumor necrosis factor α; VCAM-1, vascular cell adhesion molecule 1; VT, tidal volume; WBC, white blood cell.

## Competing interests

The authors declare that they have no competing interests.

## Authors' contributions

WW carried out the animal experiments and drafted the manuscript; FZ carried out the animal experiments; YF performed molecular biology studies; XL performed molecular biology studies; LS performed the statistical analysis; TC and HZ designed the study, and HZ revised and finalized the manuscript. All authors read and approved the final manuscript.

## Supplementary Material

Additional file 1 **Histological scoring system**. A histopathology scoring system used to analyze sections of lung, liver, kidney and small intestine. Click here for file

Additional file 2 **The sequences of primers**. The primers sequences of genes used in Real-time PCR. Click here for file
