# Supplementary material for: Glycyrrhizin protects against porcine endotoxemia through modulation of systemic inflammatory response
Source: Crit Care. 2013 Mar 11;17(2):R44. doi: 10.1186/cc12558 (PMC3672474; doi:10.1186/cc12558)
Supplement: Additional file 1 — Histological scoring system. A histopathology scoring system used to analyze sections of lung, liver, kidney and small intestine. [file cc12558-S1.DOC]

**Histological scoring system**

Lung

1. Leukocyte infiltration (leukocyte per field)

| 0 | 1 | 2 | 3 | 4 |
| --- | --- | --- | --- | --- |
| ≤10 cells | ≤25 cells | ≤50 cells | ≤75 cells | >75 cells |

2. Hemorrhage (proportion of hemorrhage areas in tissue)

| 0 | 1 | 2 | 3 | 4 |
| --- | --- | --- | --- | --- |
| not present in the field | ≤10% | ≤25% | ≤50% | >50% |

3. Alveolar wall thickness (proportion of thickened walls in all alveolar wall)

| 0 | 1 | 2 | 3 | 4 |
| --- | --- | --- | --- | --- |
| not present in the field | ≤10% | ≤25% | ≤50% | >50% |

Liver

1. Interstitial edema (proportion of enlarged septum in all septum including interlobular septa and portal tracts)

| 0 | 1 | 2 | 3 | 4 |
| --- | --- | --- | --- | --- |
| not present in the field | ≤10% | ≤25% | ≤50% | >50% |

2. Leukocyte infiltration (leukocyte per field)

| 0 | 1 | 2 | 3 | 4 |
| --- | --- | --- | --- | --- |
| ≤10 cells | ≤25 cells | ≤50 cells | ≤75 cells | >75 cells |

Kidney

1. Interstitial edema

| 0 | 1 | 2 | 3 | 4 |
| --- | --- | --- | --- | --- |
| not present in the field | sporadic occurrence | present in up to half of the field | present in up to 3/4 of the field | prevalent throughout the field |

2. Leukocyte infiltration (leukocyte per field)

| 0 | 1 | 2 | 3 | 4 |
| --- | --- | --- | --- | --- |
| ≤10 cells | ≤25 cells | ≤50 cells | ≤75 cells | >75 cells |

3. Capillary congestion

| 0 | 1 | 2 | 3 | 4 |
| --- | --- | --- | --- | --- |
| not present in the field | sporadic occurrence | present in up to half of the field | present in up to 3/4 of the field | prevalent throughout the field |

Small intestine

1. Gruenhagen spaces (proportion of Gruenhagen spaces in lumen width)

| 0 | 1 | 2 | 3 | 4 |
| --- | --- | --- | --- | --- |
| not present in the field | ≤10% | ≤25% | ≤50% | >50% |

2. Denuded Surface (proportion of denuded surfac in lumen width)

| 0 | 1 | 2 | 3 | 4 |
| --- | --- | --- | --- | --- |
| not present in the field | ≤10% | ≤25% | ≤50% | >50% |

3. Interstitial edema (proportion of edema areas in the field)

| 0 | 1 | 2 | 3 | 4 |
| --- | --- | --- | --- | --- |
| not present in the field | ≤10% | ≤25% | ≤50% | >50% |
