# Supplementary material for: Glycyrrhizin protects against porcine endotoxemia through modulation of systemic inflammatory response
Source: Crit Care. 2013 Mar 11;17(2):R44. doi: 10.1186/cc12558 (PMC3672474; doi:10.1186/cc12558)
Supplement: Additional file 2 — The sequences of primers. The primers sequences of genes used in Real-time PCR. [file cc12558-S2.DOC]

| Table S1. The sequences of primers | | |
| --- | --- | --- |
|  | Primer | |
| Gene | Forward (5’-3’) | Reverse (5’-3’) |
| HMGB1 | TTGCCGGGAGGAGCATAAGAAGAA | GTCCGCCTTTGCCATGTCTTCAAA |
| IL6 | AAACAGCAAGGAGGTACTGGCAGA | AAGCAGGTCTCCTGATTGAACCCA |
| IL10 | AAGACGTAATGCCGAAGGCAGAGA | TGCTAAAGGCACTCTTCACCTCCT |
| NF-кB p65 | AAAGACTGCCGGGATGGCTTCTAT | TTCCAGGTCCCGCTTCTTTACACA |
| ICAM-1 | TCACTGGCACAAGACTGAAGTGGA | TGGCAGAGTAGAGTGCCATCGTTT |
| VCAM-1 | AAAGACTGCCGGGATGGCTTCTAT | TTCCAGGTCCCGCTTCTTTACACA |
| PBEF | AAAGACTGCCGGGATGGCTTCTAT | TTCCAGGTCCCGCTTCTTTACACA |
| β-actin | AAGATCAAGATCATCGCGCCTCCA | ACTCCTGCTTGCTGATCCACATCT |
